# Genetic Evidence of African Slavery at the Beginning of the Trans-Atlantic Slave Trade

**DOI:** 10.1038/srep05994

**Published:** 2014-08-08

**Authors:** Rui Martiniano, Catarina Coelho, Maria Teresa Ferreira, Maria João Neves, Ron Pinhasi, Daniel G. Bradley

**Affiliations:** 1 Smurfit Institute of Genetics, Trinity College Dublin, Dublin, Ireland; 2 Life Sciences Department, University of Coimbra, Coimbra, Portugal; 3 Forensic Sciences Centre, Coimbra, Portugal; 4 Dryas Arqueologia Lda., Coimbra, Portugal; 5 Centro de Investigação em Antropologia e Saúde, Coimbra, Portugal; 6 School of Archaeology, University College Dublin, Dublin, Ireland; 7 UCD Conway Institute of Biomolecular and Biomedical Research, University College Dublin, Dublin, Ireland

## Abstract

An archaeological excavation in Valle da Gafaria (Lagos, Portugal), revealed two contiguous burial places outside the medieval city walls, dating from the 15^th^–17^th^ centuries AD: one was interpreted as a Leprosarium cemetery and the second as an urban discard deposit, where signs of violent, unceremonious burials suggested that these remains may belong to slaves captured in Africa by the Portuguese. We obtained random short autosomal sequence reads from seven individuals: two from the latter site and five from the Leprosarium and used these to call SNP identities and estimate ancestral affinities with modern reference data. The Leprosarium site samples were less preserved but gave some probability of both African and European ancestry. The two discard deposit burials each gave African affinity signals, which were further refined toward modern West African or Bantu genotyped samples. These data from distressed burials illustrate an African contribution to a low status stratum of Lagos society at a time when this port became a hub of the European trade in African slaves which formed a precursor to the transatlantic transfer of millions.

Recent archaeological excavations in an area outside the mediaeval walls of Lagos city in southern Portugal (Valle da Gafaria) revealed two adjacent burial places with unusual inhumation patterns^1^,^2^; both dated to the 15th–17th centuries. The first of these has been interpreted as a burial site attached to a leprosarium which would have been traditionally situated outside urban limits, and the eleven individuals recovered from this necropolis exhibited several pathological lesions both in the skull and postcranial skeleton^3^. Leprosy was diagnosable in two of these individuals and it is expected that people suffering from a range of diseases were also housed in such a context^3^.

The second comprised an urban discard deposit (UDD) where skeletal remains belonging to 158 individuals including males and females, adults and sub-adults, were retrieved. These were distressed burials; the bodies were found together with urban and domestic garbage in a large pit with apparent disrespect for the canonical burial traditions. It was possible to infer that these individuals were deposited in the trash dump area (both in the sinkhole and in its boundaries) and immediately covered with trash deposits. Many were deposited in atypical positions, suggesting a pronounced lack of care in inhumation. Also both direct and indirect cases of violence were recorded; for example, three cases of hand and/or feet binding^1^.

Interestingly, cultural items associated with some skeletons (beads, ivory and bone sculptures)^2^, and intentional dental modifications suggested sub Saharan African origins for some of the individuals in the pit^1^. Historical sources document African slave capture and commerce by Portuguese merchants since the 15th century and a human sample from the urban discard deposit yielded a radiocarbon dated of cal. AD 1420–1480^1^,^2^.

In this study we use next generation sequencing (NGS) of historical DNA sampled from bones of seven individuals from these two sites to estimate ancestry, sex and DNA preservation.

Results confirm African ancestry in two samples from the urban discard deposit. The Leprosarium site revealed a diverse ancestral composition, with suggestion of both European and African, or African-admixed ancestry, but with less certainty due to lower preservation and genomic coverage. Lagos was one of the most important harbours in the Iberian Peninsula, a hub of the early African slave trade within Europe, and the burials analysed here may be among the earliest victims of a tragic commerce that subsequently amplified to millions of forced transatlantic transfers.

## Results

### Sequencing results

We extracted DNA from nine bone samples from skeletons exhumed from Valle da Gafaria site in Lagos, Portugal. DNA extracts were then incorporated into NGS libraries^4^, amplified with distinct indexes and pooled in equimolar content with 18 samples from other experiments. A partial MiSeq run yielded ~5.9 million reads containing indexes corresponding to the libraries prepared with the samples from the UDD and the Leprosarium cemetery. We trimmed adapter sequences and aligned the reads using *BWA*^5^ to the Human Reference Genome (hg19), filtering for base (q ≥ 15) and mapping quality (q ≥ 30), removing paralogs and selecting only uniquely aligned reads. We removed duplicate reads, which consisted of less than 0.25% across all samples. After read filtration we kept ~200 thousand reads mapping to the human reference genome in total.

The bone samples with highest endogenous human DNA content were Individuals 125 and 166 (7.6% and 9.99% respectively), both from the UDD site, and five other samples yielded human DNA at levels around 1% (0.57–1.88%) (Table 1). The lowest endogenous contents observed were for Individuals 25 and 65 (UDD), with 0.10% and 0.17%, and because of reduced numbers of reads after filtration (799 and 1657 respectively) these samples were removed from further analyses.

### Cytosine deamination patterns

**Figure 1:**
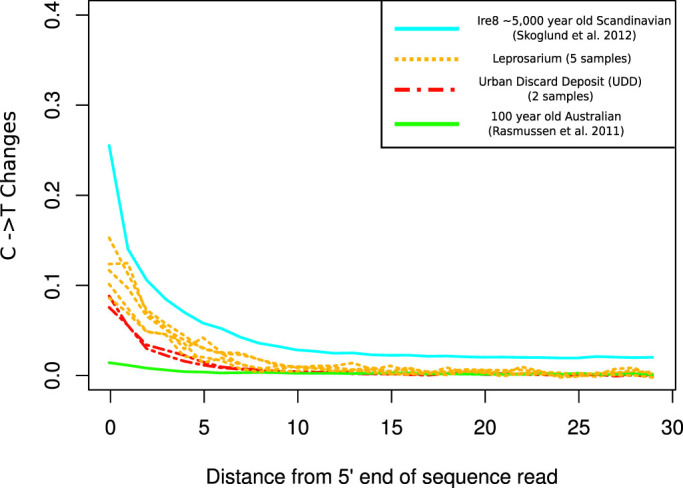
Comparison of Cytosine Deamination patterns in the 5′ end of sequencing reads between the samples from the Leprosarium (yellow dotted lines) and UDD (red dotted lines) burial sites with a ~5,000 year old Scandinavian hunter-gatherer and a 100 year old Australian Aboriginal. The range of C to T changes suggest the authenticity of the data.

Cytosine deamination at the 5′-end of DNA fragments (leading to C to T changes) is a signal of postmortem chemical degradation suggesting authenticity in ancient DNA sequence^6^ and has been shown to increase with time^7^. We used *PMDtools*^8^ to examine these changes in our samples and compared patterns with those present in published data (Figure 1). Cytosine deamination values observed across all samples are intermediate (0.8–0.15) when compared to the very low (0.02) and very high (0.26) fraction of C->T changes respectively in a 100 year old Australian aboriginal^9^ and a 5,000 year old Neolithic Scandinavian^10^, as would be expected from the age estimations for this burial site. The mean fraction of reads containing cytosine deamination appears to be slightly lower for the two high endogenous DNA samples belonging to the UDD site (0.09) than that within reads from the five moderately preserved Leprosarium samples (0.12).

### Sex estimation

**Figure 2:**
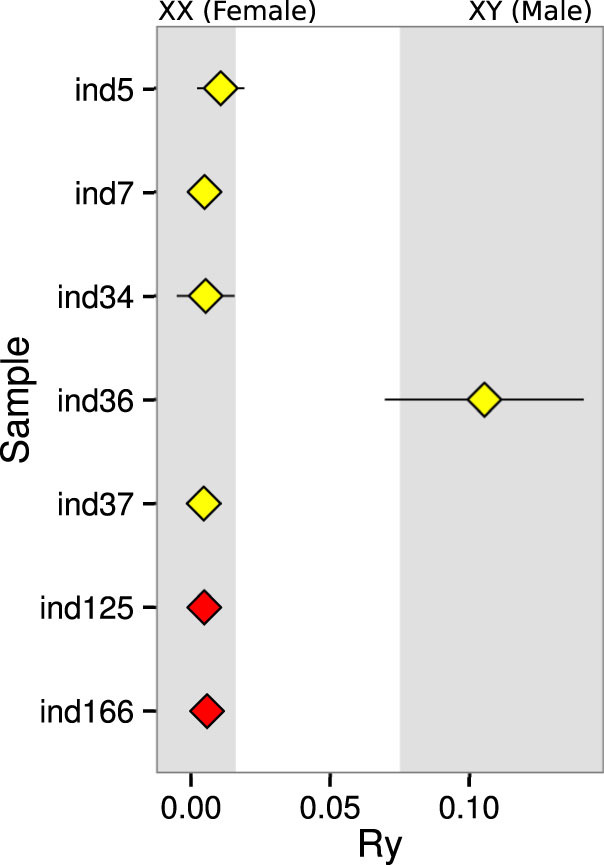
Sex identification based on shotgun sequencing data. Ry - Ratio of the number of reads aligned to the Y-chromosome divided by the sum of the number reads aligned to the Y- and X-chromosomes. Gray shaded areas represent threshold for acceptance of assignment, calibrated with modern and ancient genomes^11^. Error bars correspond to 95% confidence intervals.

Sex estimation can sometimes be crucial to understand certain archaeological contexts and it can be a very challenging task for anthropologists, especially when dealing with young individuals, bone degradation or absence of more sexually dimorphic bones such as the pelvis. A recently published method^11^, calibrated with modern and ancient sequence data, has shown that it is possible to confidently obtain this information by estimating the fraction of reads that map to the X- and Y-chromosomes. We applied this method to our data (Figure 2 and Supplementary Table S1) and determined that all samples were female, with the exception of Individual 36. Confidence intervals of sex determination for Sample 5 and 36 overlap the stringent boundaries of certainty (Female if Ry > 0.016, Male if Ry < 0.075, represented by gray shaded areas), but more sequence data would likely reveal that these assignments are correct.

### Population genetics analysis

Despite the very low coverage (less than 0.002% of the genome, Table 1) across all samples, we still obtained sufficient overlap with known SNP positions genotyped in a range of worldwide population samples, allowing us to perform principal component analysis (PCA) to assess population affinities (the numbers of genotypes identified in the samples analysed is shown in Table 1). We applied Procrustes transformation^10^ to merge individual, non-overlapping SNP data of the samples analysed in the present work into a single PCA plot.

**Figure 3:**
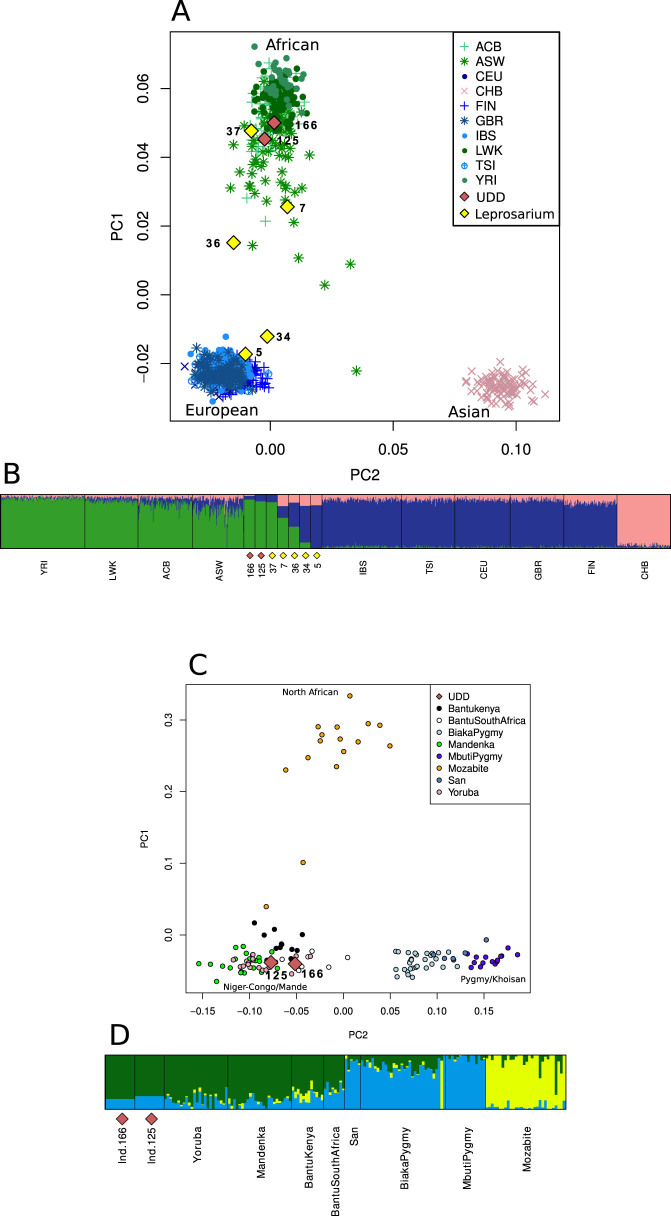
(A) Procrustes transformation of Principal Component Analysis combining UDD (lozenges in red) and Leprosarium (lozenges in yellow) samples merged with selected African, European and Asian populations from the 1000 Genomes Omni dataset. (B) ADMIXTURE Plot of the same data used for the PCA assuming k = 3. Population key: ACB, African Caribbeans in Barbados; ASW, Americans of African ancestry in SW USA; CEU, Utah residents (CEPH) with Northern and Western European ancestry; CHB, Han Chinese in Beijing, China; FIN, Finnish in Finland; GBR, British in England and Scotland; IBS, Iberian population in Spain; LWK, Luhya in Webuye, Kenya; TSI, Toscani in Italy; YRI, Yoruba in Ibadan, Nigeria. (C) Procrustes transformation of Principal Component Analysis combining both individuals from UDD (125 and 166, lozenges in red) merged with African samples from the HGDP dataset. (D) ADMIXTURE Plot of the same data used for the PCA assuming k = 3.

In the first instance, we merged our data with the 1000 Genomes project dataset (Omni) (Figure 3a), which consists of high density genotype data of worldwide populations^12^, which allowed us to observe a positioning of the samples sequenced in the present study toward distinct continental clusters (Figure 3a). PCA revealed a greater affinity of samples 7, 36 and 37 (Leprosarium) and samples 125 and 166 (UDD) with African (LWK, YRI), African ancestry (ASW) and African Caribbean (ACB) populations. Individuals 5 and 34 (Leprosarium) fell adjacent to the European population cluster (IBS, GBR, TSI, FIN and CEU). Separate PCA plots are shown in Supplementary Figure S1. Interestingly, the Leprosarium individuals 7 and 36 were placed near the edge of populations of African ancestry, along with individuals from the Caribbean and America where substantial partial European admixture is well documented, suggesting the possibility of African-European admixture.

*ADMIXTURE*^13^, an unsupervised model-based method, was employed to estimate ancestral fractions of UDD and Leprosarium samples. We performed 10 repeated runs assuming *k* clusters from 2 to 10 ancestral populations and cross-validation errors pointed to an optimal *k* = 3 (Supplementary Figure S5a). *CLUMPP*^14^ was used to average clustering results between samples and obtain the best configuration of clusters across datasets. These results were plotted with *distruct*^15^ (Figure 3b and Supplementary Figure S2). This analysis confirms the results obtained with the PCA, and also suggests possible African-European admixture for Individuals 7 and 36.

To further investigate population affinity we merged our data with the less densely genotyped data from the Human Genome Diversity Project^16^ but which contains a wider sampling of African populations. We focused on the two samples from the UDD (125 and 166) with superior levels of endogenous DNA and greater than 500 SNP calls. The principal component analysis defined loose population clusters that correspond to three African groups: North African Mozabites; Khoisan and Pygmy populations; a grouping of Niger-Congo speaking populations (Figure 3c). The two historical samples clearly clustered within the latter, which included Kenyan and South African Bantu populations plus West African Yoruba and Mandenka samples. When performing ADMIXTURE analysis, although a lower cross-validation error is obtained at k = 2 (Supplementary Figure S5b), this affinity becomes evident at k = 3 (Figure 3d). Separately displayed PCA (Supplementary Figure S3) and ADMIXTURE analyses (Supplementary Figure S4) for Leprosarium and UDD samples merged with the HGDP dataset are consistent.

### Investigation of modern human contamination

We investigated the possibility of population affinity signals resulting from modern human contamination using PMDtools^8^ to compare analysis using only sequence reads with evidence of deamination (a modification prevalent in ancient DNA) to those of unfiltered data. In PCAs, as a result of the data reduction by filtration for deamination, complete separation of African and European reference clusters was not apparent, preventing confirmation of ancestral affinity for the less-preserved samples from the Leprosarium. Therefore, inference from these samples must carry a degree of caution. However, this analysis did have sufficient resolution to confirm African ancestry of samples 125 and 166 from the discard deposit (Supplementary Figure S6). Attribution of female sex to samples 7, 125 and 166 using only deaminated reads agreed with the previous analysis (Comparison of both analysis is shown in Supplementary Figure S7 and values obtained are shown in Supplementary Table S2). All other samples were left with under 1000 filtered reads which were insufficient to provide accurate estimates.

### mtDNA haplogroup assignment

After aligning the filtered Next-Generation Sequencing Reads to the revised Cambridge Reference Sequence (rCRS)^17^, we obtained a low number of reads covering informative sites and we were not able to identify complete haplotypes. Nonetheless, we observe certain mutations in our data that point to the probability of African mtDNA lineages being present in the five samples (Supplementary Table S3) that have shown a greater affinity with African Populations in PCA and ADMIXTURE analysis. These were L3 sub lineages in samples 166 and 36; L3′4′6 in 37; L2b1 in sample 125 and L1b1 in sample 7.

## Discussion

In this work we use low coverage next-generation sequencing data to address common issues encountered in archaeological contexts: sex estimation, sample preservation and population affinity. By pooling several samples in the same sequencing run and obtaining just a few tens of thousands sequencing reads per sample, we have estimated the sex of seven out of nine samples sequenced. Furthermore, we characterized cytosine deamination patterns within the data and conclude that they are broadly consistent with those expected from historical bone samples. The modest SNP data obtained for these samples was sufficient to perform PCA and ADMIXTURE analyses, tentatively suggesting a diversity of ancestral backgrounds in the Leprosarium cemetery, with two individuals presenting a greater similarity with modern European (5 and 34) and three with a degree of African ancestry (7, 36, 37). The latter seems less likely to have resulted from post-mortem contamination but the limited extent of these data were such that this may not formally be excluded. The superior preservation of the two African individuals (125 and 165) from the urban discard deposit burial site and resistance of their genetic signals to restriction of the data set to reads with showing deaminations encouraged investigation of their origins at a finer detail, showing affinity with Bantu-speaking groups and Western African Mandenka and Yoruba populations, as expected from historical records. A previous study^18^ has shown that African chromosomal segments in African-American individuals were most similar to Niger-Kordofanian-speaking populations such as those included here. Here, we did not achieve sufficient resolution in PCA and ADMIXTURE to distinguish between Bantu and non-Bantu Niger-Kordofanian populations. Mitochondrial DNA coverage was insufficient to ascertain complete haplotypes, but the base calls at polymorphisms that define sub lineages of haplogroups L1, 2 and 3 corroborate PCA and ADMIXTURE results and are compatible with the lineages identified in a previous study in an African slave cemetery in Brazil^19^. Future studies addressing the Atlantic Slave Trade would benefit immensely from publicly available dense genotypes or high coverage genomes from regions where the trade was most intense, such as Mauritania, Guinea, Senegal, Sierra Leone, Gambia, Angola and Mozambique. The evidence of African ancestry presented here, coupled with archaeology supporting these origins, violence and distressed burial suggests that the urban discard deposit human remains belonged to slaves brought to Maritime 15th/17th century Portugal; an early snapshot of the ignominious commerce that will become the Atlantic slave trade.

## Methods

### Sample preparation and DNA extraction

Sample cleaning, drilling, extraction and library preparation were performed in clean-room facilities at the Ancient DNA lab, Smurfit Institute, Trinity College Dublin (Ireland) which is exclusively dedicated to aDNA sample processing. Blank controls were incorporated in extractions, library preparation and PCR reactions to monitor the possibility of contamination. Bones were decontaminated by UV light exposure and by removing its surface using a Dremel drill. The densest portion for the different bones was sawed off using a circular saw, and drilled until becoming powder. Extractions of 0.2 grams of bone powder for each sample were performed^20^ with modifications described elsewhere^21^. Undigested pellets were re-extracted^22^, purified with Minelute columns (Qiagen MinElute PCR Purification Kit, Qiagen, Hilden, Germany) and the resulting eluate was used for library preparation.

### Library preparation method

A published Illumina sequencing library preparation method^4^ was used to incorporate DNA fragments into Next-Generation Sequencing adaptors with the following changes: we used T4 DNA polymerase buffer (Thermo Scientific) instead of Tango buffer in the Blunt-End Repair step; replaced Solid Phase Reversible Immobilization (SPRI) purification with Minelute Purification, and instead of the final purification after Adapter Fill-in we heat inactivated Bst Polymerase by incubating the libraries for 20 minutes at 80°C.

Libraries were amplified for 12 cycles in a separate room used only for PCR of ancient DNA, with AccuPrime Pfx Polymerase (Invitrogen) and a different indexing oligo^4^ for each sample so that multiple samples can be pooled and sequenced in the same sequencing run. With the purpose of screening for ancient DNA content, we pooled 27 samples of different origins and time periods, 9 of which belonged to the burial site in Lagos, Portugal. The resulting PCR product was purified with Minelute spin columns and eluted in 20 ul EB buffer. After DNA quantification using Quant-iT dsDNA HS Assay kit (Invitrogen, Oregon, USA) and with Agilent 2100 Bioanalyzer High Sensitivity DNA kit. Samples were pooled in equimolar concentrations by averaging the values obtained by both quantification methods and sequenced for 50 cycles, single-end reads mode, in a Illumina MiSeq instrument located in the Institute of Molecular Medicine (IMM), Trinity College Dublin, along with PhiX control at 1%.

### Read alignment and filtering

Cutadapt^23^ was used to trim adaptor sequences present in the raw reads. These reads were then aligned to the human reference genome (USCS hg19) using BWA^5^. The alignment parameters were set in a way that low quality bases (“-q 15”) were trimmed from the 3′ end of the reads and seeding was disabled to improve accuracy (“- l 1024”)^24^. We then excluded reads with mapping quality inferior to 30 (“-q 30”) and removed duplicates using SAMtools^25^. We also selected reads containing the SAMtools flag X1 to filter for paralogs. The resulting bam file containing only confidently mapped reads was used to estimate the percentage of endogenous DNA present in the ancient samples by comparing the fraction of mapped reads with the total number of reads obtained with same barcode (Table 1).

### Sample preservation and deamination patterns

To compare cytosine deamination patterns in the samples analysed in the present work, we downloaded sequence reads of a ~7,000 year-old Scandinavian Hunter-Gatherer^10^ and a 100 year-old Australian Aboriginal^9^ and aligned to the hg19 human reference genome with the same filtering criteria used for our data. After downsampling aligned reads to 100,000 (to match the number of aligned reads in Individual 166), we characterized cytosine deamination patterns using PMDtools^8^ and the results were plotted with a customized *PMDtools* R script.

### Sex determination

A recently developed method for sex determination using NGS reads^11^ was employed in our samples using confidently aligned reads filtered as above. Results are presented in Figure 2.

### Principal component analysis

In order to compare our ancient samples to datasets of modern human populations, we identified bases in known SNP positions using Genome Analysis Tool Kit (GATK) in Pileup mode by providing an interval file (“-L snps.bed”) for each modern human genotype dataset. Specifically, we used the 1000 Genomes dataset (ftp.1000genomes.ebi.ac.uk/vol1/ftp/technical/working/20120131_omni_genotypes_and_intensities/) and genotypes from the Human Genome Diversity Project (HGDP; http://www.hagsc.org/hgdp/) flipped to hg19 strand orientation. For Principal Component and admixture analysis we filtered our data in a similar way as described previously^10^. Briefly, we only included reads with base and mapping quality of a least 15 and 30, respectively. Potentially false mutations that may have been originated by cytosine deamination (C to T and G to A) were excluded from analysis and SNP data was converted to *PLINK* format files^26^. Because of the very low coverage of the data obtained, the vast majority of positions in the genome are covered by a single read only and, therefore, all genotypes were converted to homozygous. In the case of a chromosomal position being covered by more than one read only a nucleotide base was randomly chosen and included in the analysis. Likewise, and to avoid any bias in population variability, all heterozygous positions in human diversity datasets were converted to homozygous by randomly picking one of the alleles. PLINK was used to merge the ancient samples genotype data with Omni and HGDP datasets separately. Principal Component Analysis was done using *SMARTPCA*^27^, removing SNPs in LD with nearby SNPs (r-squared > 0.2). We used the R package “vegan” (http://cran.r-project.org/web/packages/vegan/index.html) to do Procrustes transformation on the Principal Component coordinates. Because of the high-density genotyping in the Omni dataset, we were able to retrieve sufficient positions to perform Procrustes transformation on all ancient samples (Figure 3a). For the HGDP dataset, the number of SNPs called is too low in the majority of samples to provide a clear clustering between populations. Therefore, for Procrustes transformation of the HGDP merged with historical DNA genotypes, we picked our two best samples in terms of endogenous DNA and number SNP positions identified (166 and 125) (Figure 3c). Principal Component Analysis was plotted with R version 2.14.1^28^.

### Estimation of modern human contamination

To access whether our data is being affected by modern human contamination, we used PMDtools to extract reads with evidence of deamination (PMD score 3) and compared Principal Component Analysis and sex determination using these reads with unfiltered data (PMD score -2).

### Model-based clustering

Using the aforementioned filtered datasets, we performed ADMIXTURE^13^ runs for values of k (ancestral populations) ranging from 2 to 10. Each run of ADMIXTURE for k = 2 to 10 has a coefficient variation (CV) value associated to it. CLUMPP^14^ version 1.1.2 was used to average clustering results between samples and obtain the best configuration of clusters across datasets with the *fullSearch* algorithm. Clustering results were visualised with distruct^15^ and are presented in Figure 3b and 3d.

### Tentative mitochondrial DNA haplogroup identification

In order to identify mitochondrial DNA (mtDNA) haplogroups, we selected reads at least 25 bp long and used *seqtk* (https://github.com/lh3/seqtk) to trim the first and last 3 bp in sequencing reads to minimize the effect of deamination and sequencing errors. Reads were aligned to the revised Cambridge Reference Sequence (rCRS) and filtered as above. The final bam file was uploaded to MitoBamAnnotator^29^ which identifies mtDNA mutations which are then analysed with HaploGrep^30^ identifying the most likely haplogroup to which a sample belongs.

## Author Contributions

R.M., D.B. and R.P. conceived and designed the study. R.M. performed the experiments and data analysis. M.T.F. and M.J.N. excavated the skeletal human remains; M.T.F., C.C. and M.J.N. made the anthropological study of the skeletons. All authors contributed to the manuscript preparation.

## Additional Information

**Accession codes**: Sequence reads were uploaded to the Sequence Read Archive at the ENA (European Nucleotide Archive), study accession PRJEB6056.

## Supplementary Material

Supplementary Information SUPPLEMENTARY INFO

## Figures and Tables

**Table 1:** Number of sequencing reads before and after alignment and filtering for base (q ≥ 15), mapping quality (q ≥ 30) and duplicate removal. Sex estimation results and genotyped SNPs in Omni and HGDP datasets. UDD – Urban discard deposit

Burial Context	Sample	Bone sampled	Total Reads	Hits to hg19*	Fraction of Human Reads (%)	Genome Coverage (x)	Sex Determination**	Genotyped SNPs*	
								Omni	HGDP
Leprosarium	Ind. 5	metatarsal	703096	10740	1.53	0.00018	XX	525	156
Leprosarium	Ind. 7	metatarsal	866232	16300	1.88	0.00027	XX	762	208
Leprosarium	Ind. 34	Left Fibula	616433	3800	0.62	0.00006	XX	191	50
Leprosarium	Ind. 36	metatarsal	595411	9502	1.6	0.00016	XY	390	102
Leprosarium	Ind. 37	4th lumbar vertebra	1459865	8255	0.57	0.00014	XX	383	94
UDD	Ind. 25 ‡	metatarsal	766371	799	0.1	0.00001	n/a	32	12
UDD	Ind. 65 ‡	metatarsal	960013	1657	0.17	0.00002	n/a	60	14
UDD	Ind. 125	metatarsal	597681	45414	7.6	0.00077	XX	2131	551
UDD	Ind. 166	metatarsal	1076124	107507	9.99	0.00182	XX	4961	1331

*Reads were filtered by base quality ≥ 15 and mapping quality ≥ 30. Duplicate reads were excluded and only unambiguously mapped reads were kept.

**Sex determination method^11^. Results shown in more detail in Supplementary Table S1.

^‡^Individuals removed from posterior analysis because of insufficient endogenous DNA read number.

## References

[b1] Neves M., Almeida M. & Ferreira M. Separados na vida e na morte: retrato do tratamento mortuário dado aos escravos africanos na cidade moderna de Lagos. XELB Rev. 10, 547–560 (2010).

[b2] Neves M., Almeida M. & Ferreira M. História de um arrabalde durante os séculos XV e XVI: o “poço dos negros” em Lagos (Algarve, Portugal) e o seu contributo para o estudo dos escravos em Portugal. in A Herança do Infante 29–46 (2011).

[b3] Ferreira M. T., Neves M. J. & Wasterlain S. N. Lagos leprosarium (Portugal): evidences of disease. J. Archaeol. Sci. 40, 2298–2307 (2013).

[b4] Meyer M. & Kircher M. Illumina sequencing library preparation for highly multiplexed target capture and sequencing. Cold Spring Harb. Protoc. (2010) 10.1101/pdb.prot5448 20516186

[b5] Li H. & Durbin R. Fast and accurate short read alignment with Burrows-Wheeler transform. Bioinformatics 25, 1754–60 (2009). 19451168 10.1093/bioinformatics/btp324 PMC2705234

[b6] Brotherton P. *et al.* Novel high-resolution characterization of ancient DNA reveals C > U-type base modification events as the sole cause of post mortem miscoding lesions. Nucleic Acids Res. 35, 5717–28 (2007). 17715147 10.1093/nar/gkm588 PMC2034480

[b7] Sawyer S., Krause J., Guschanski K., Savolainen V. & Pääbo S. Temporal patterns of nucleotide misincorporations and DNA fragmentation in ancient DNA. PLoS One 7, (2012). 10.1371/journal.pone.0034131 PMC3316601 22479540

[b8] Skoglund P. *et al.* Separating endogenous ancient DNA from modern day contamination in a Siberian Neandertal. Proc. Natl. Acad. Sci. 1318934111– (2014). 10.1073/pnas.1318934111. 10.1073/pnas.1318934111 PMC3926038 24469802

[b9] Rasmussen M. *et al.* An Aboriginal Australian Genome Reveals Separate Human Dispersals into Asia. Sci. 334, 94–98 (2011). 10.1126/science.1211177 PMC3991479 21940856

[b10] Skoglund P. *et al.* Origins and Genetic Legacy of Neolithic Farmers and Hunter-Gatherers in Europe. Science (80-.). 336, 466–469 (2012). 10.1126/science.1216304 22539720

[b11] Skoglund P., Storå J., Götherström A. & Jakobsson M. Accurate sex identification of ancient human remains using DNA shotgun sequencing. J. Archaeol. Sci. 40, 4477–4482 (2013).

[b12] The 1000 Genomes Project Consortium. An integrated map of genetic variation. Nature 135, 0–9 (2012). 10.1038/nature11632 PMC3498066 23128226

[b13] Alexander D. H., Novembre J. & Lange K. Fast model-based estimation of ancestry in unrelated individuals. Genome Res. 19, 1655–64 (2009). 19648217 10.1101/gr.094052.109 PMC2752134

[b14] Jakobsson M. & Rosenberg N. A. CLUMPP: a cluster matching and permutation program for dealing with label switching and multimodality in analysis of population structure. Bioinformatics 23, 1801–1806 (2007). 17485429 10.1093/bioinformatics/btm233

[b15] Rosenberg N. A. DISTRUCT: a program for the graphical display of population structure. Mol. Ecol. Notes 4, 137–138 (2004).

[b16] Li J. Z. *et al.* Worldwide human relationships inferred from genome-wide patterns of variation. Science 319, 1100–4 (2008). 18292342 10.1126/science.1153717

[b17] Andrews R. M. *et al.* Reanalysis and revision of the Cambridge reference sequence for human mitochondrial DNA. Nat. Genet. 23, 147 (1999). 10508508 10.1038/13779

[b18] Bryc K. *et al.* Genome-wide patterns of population structure and admixture in West Africans and African Americans. Proc. Natl. Acad. Sci. U. S. A. 107, 786–791 (2010). 20080753 10.1073/pnas.0909559107 PMC2818934

[b19] Jaeger L. H., de Souza S. M. F. M., Dias O. F. & Iñiguez A. M. Mycobacterium tuberculosis complex in remains of 18th-19th century slaves, Brazil. Emerg. Infect. Dis. 19, 837–9 (2013). 23697340 10.3201/eid1905.120193 PMC3647487

[b20] Yang D. Y., Eng B., Waye J. S., Dudar J. C. & Saunders S. R. Technical note: improved DNA extraction from ancient bones using silica-based spin columns. Am. J. Phys. Anthropol. 105, 539–543 (1998). 9584894 10.1002/(SICI)1096-8644(199804)105:4<539::AID-AJPA10>3.0.CO;2-1

[b21] MacHugh D. E., Edwards C. J., Bailey J. F., Bancroft D. R. & Bradley D. G. The Extraction and Analysis of Ancient DNA From Bone and Teeth: a Survey of Current Methodologies. Anc. Biomol. 3, 81 (2000).

[b22] Orlando L. *et al.* Recalibrating Equus evolution using the genome sequence of an early Middle Pleistocene horse. Nature 499, 74–8 (2013). 23803765 10.1038/nature12323

[b23] Martin M. Cutadapt removes adapter sequences from high-throughput sequencing reads. EMBnet.journal 17, pp. 10–12 (2011).

[b24] Schubert M. *et al.* Improving ancient DNA read mapping against modern reference genomes. BMC Genomics 13, 178 (2012). 22574660 10.1186/1471-2164-13-178 PMC3468387

[b25] Li H. *et al.* The Sequence Alignment/Map (SAM) Format and SAMtools 1000 Genome Project Data Processing Subgroup. Bioinformatics 25, 2078–2079 (2009). 19505943 10.1093/bioinformatics/btp352 PMC2723002

[b26] Purcell S. *et al.* PLINK: a tool set for whole-genome association and population-based linkage analyses. Am. J. Hum. Genet. 81, 559–575 (2007). 17701901 10.1086/519795 PMC1950838

[b27] Patterson N., Price A. L. & Reich D. Population structure and eigenanalysis. PLoS Genet. 2, 2074–2093 (2006). 10.1371/journal.pgen.0020190 PMC1713260 17194218

[b28] R Development Core Team, R. R: A Language and Environment for Statistical Computing. R Found. Stat. Comput. 1, 409 (2011).

[b29] Zhidkov I., Nagar T., Mishmar D. & Rubin E. MitoBamAnnotator: A web-based tool for detecting and annotating heteroplasmy in human mitochondrial DNA sequences. Mitochondrion 11, 924–928 (2011). 21875693 10.1016/j.mito.2011.08.005

[b30] Kloss-Brandstätter A. *et al.* HaploGrep: a fast and reliable algorithm for automatic classification of mitochondrial DNA haplogroups. Hum. Mutat. 32, 25–32 (2011). 20960467 10.1002/humu.21382

