# Supplementary material for: Genetic Evidence of African Slavery at the Beginning of the Trans-Atlantic Slave Trade
Source: Sci Rep. 2014 Aug 8;4:5994. doi: 10.1038/srep05994 (PMC4125989; doi:10.1038/srep05994)
Supplement: Supplementary Information — SUPPLEMENTARY INFO [file srep05994-s1.pdf]

# Supplementary Information

## Genetic Evidence of African Slavery at the Beginning of the Trans-Atlantic Slave Trade

Rui Martiniano[1], Catarina Coelho[2], Maria Teresa Ferreira[2,3,5], Maria João Neves[2,4,5], Ron Pinhasi*[6,7] & Daniel G. Bradley*[1]

1 – Smurfit Institute of Genetics, Trinity College Dublin, Dublin, Ireland

2 - Life Sciences Department, University of Coimbra, Coimbra, Portugal

3 - Forensic Sciences Centre, Coimbra, Portugal

4 - Dryas Arqueologia Lda., Coimbra, Portugal

5- Centro de Investigação em Antropologia e Saúde,Coimbra, Portugal

6 - School of Archaeology, University College Dublin, Dublin, Ireland

7 – UCD Conway Institute of Biomolecular and Biomedical Research, University College Dublin, Dublin, Ireland

*corresponding authors

# Supplementary Figure S1

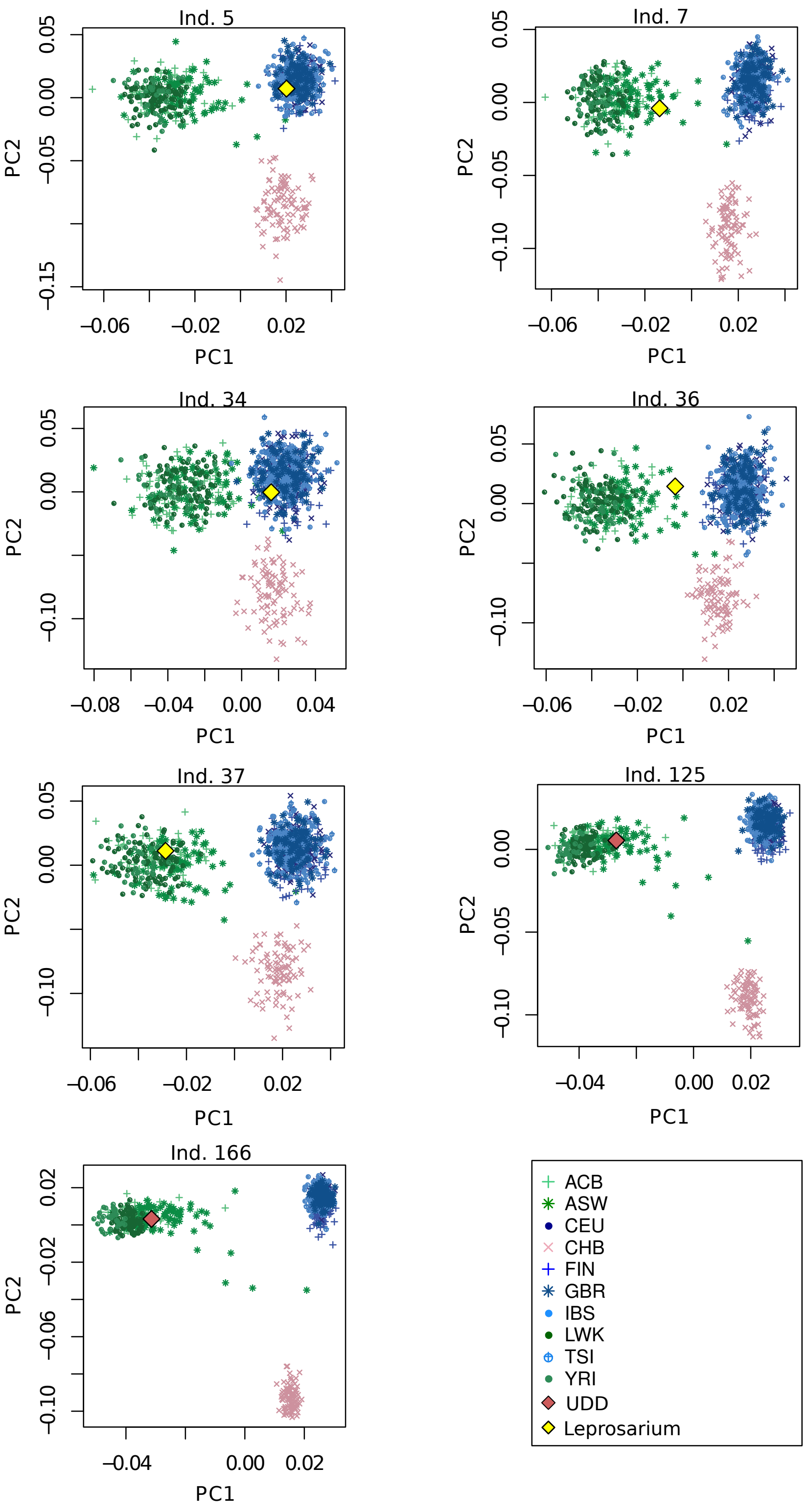


**Supplementary Figure S1** - Principal Component Analysis[1] of genotypes belonging to the samples from the urban discard deposit (lozenges in red) and Leprosarium (lozenges in yellow) merged separately with 1000 Genomes populations[2].

# Supplementary Figure S2

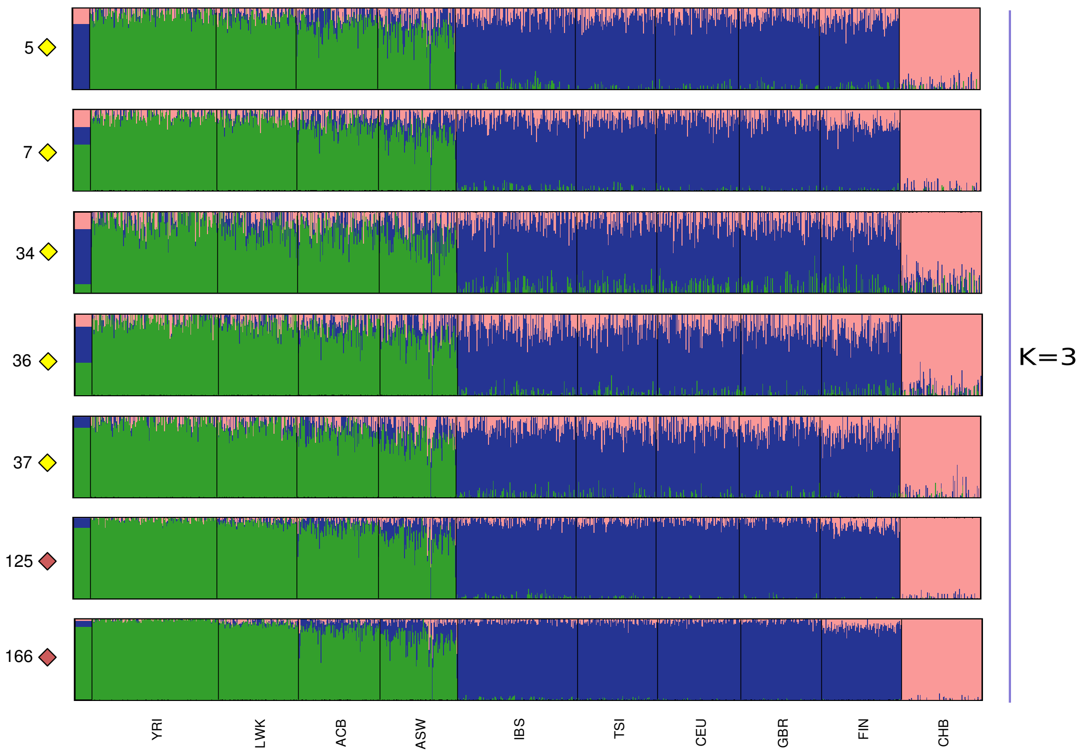


**Supplementary Figure S2** - ADMIXTURE[3] runs assuming 3 clusters for each sample from the urban discard deposit (lozenges in red) and Leprosarium (lozenges in yellow) merged separately with the 1000 Genomes populations genotype data[1].

# Supplementary Figure S3

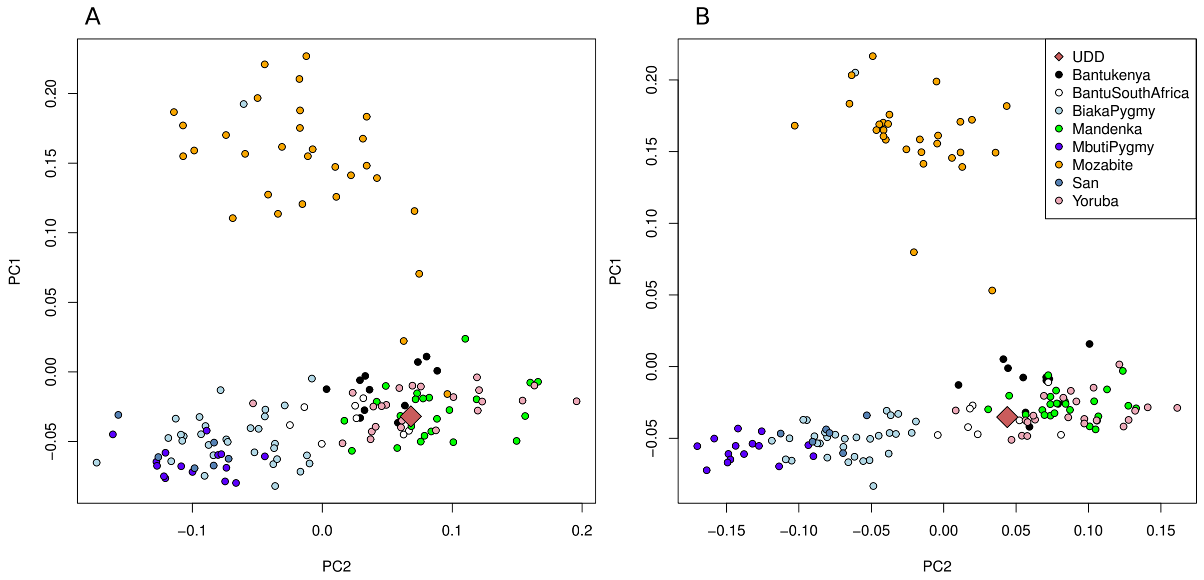


**Supplementary Figure S3** - Principal Component Analysis[1] of the samples from the urban discard deposit (UDD) (lozenges in red) merged with African populations from the HGDP dataset[4]. **A)** Sample 125. **B)** Sample 166.

# Supplementary Figure S4

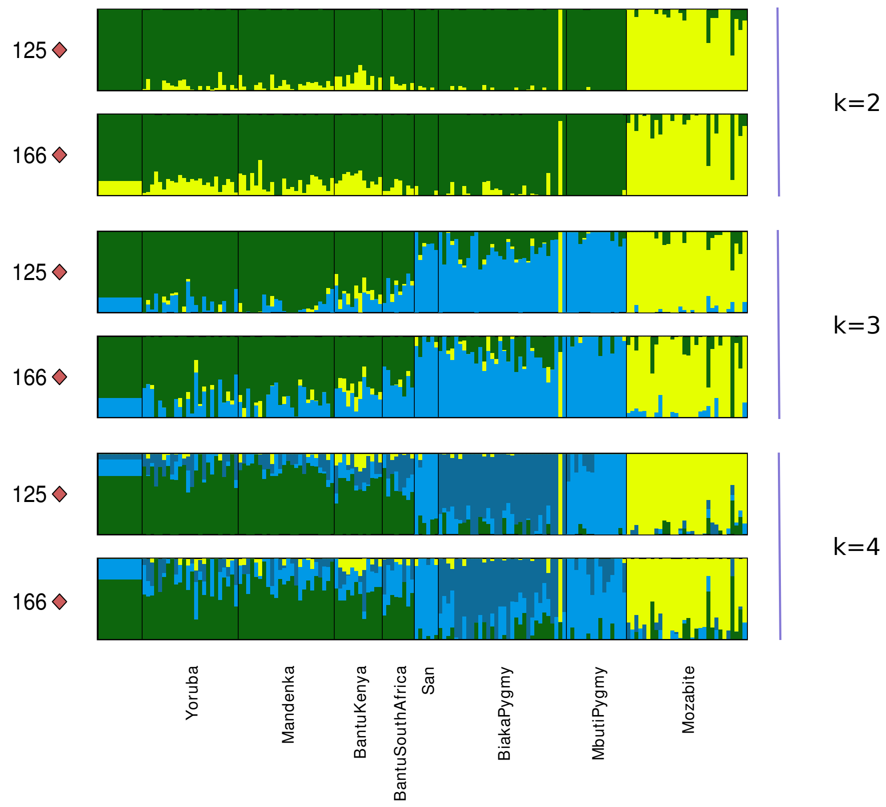


**Supplementary Figure S4** - ADMIXTURE[3] runs assuming 2, 3 and 4 clusters for samples 125 and 166 from the urban discard deposit site (lozenges in red) merged separately with African populations from the HGDP dataset[4].

# Supplementary Figure S5

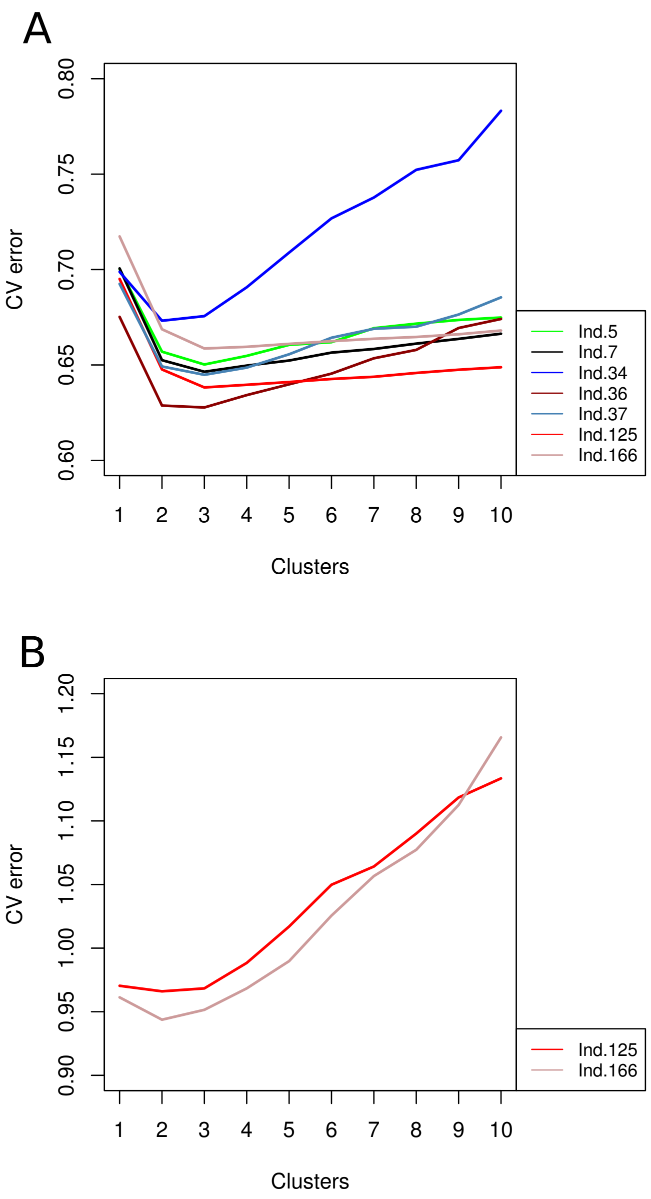


**Supplementary Figure S5** - Cross-validation (CV) errors of ADMIXTURE[3] runs for **A)** each one of the urban discard deposit and leprosarium samples merged with 1000 Genomes dataset[2] and **B)** Urban discard deposit samples 125 and 166 merged with African populations from the HGDP dataset[4].

# Supplementary Figure S6

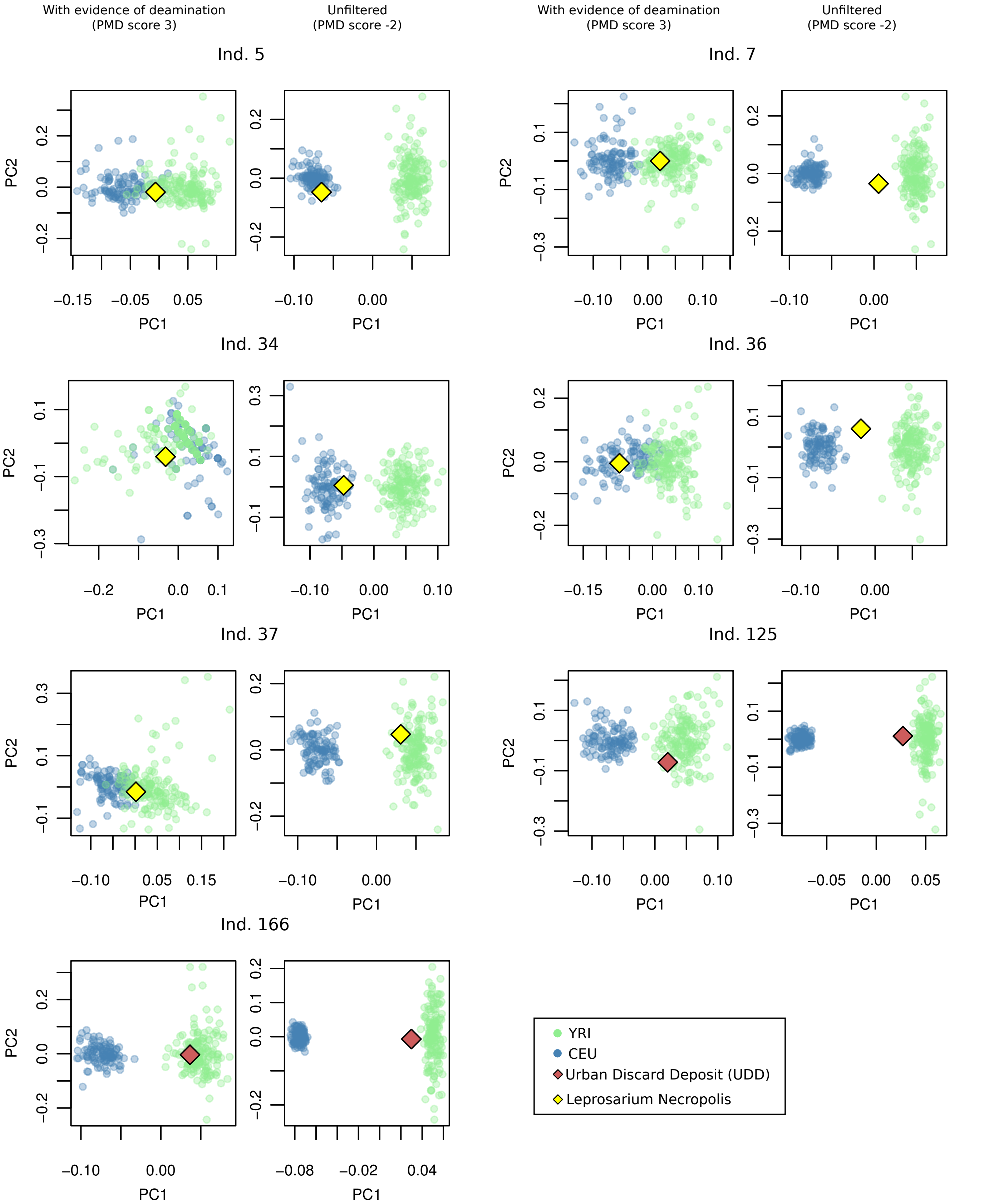


**Supplementary Figure S6** - Principal Component Analysis[1] of UDD and Leprosarium samples SNP data merged individually with 1000 Genomes[2] genotypes from Yoruba (YRI) and Northern Europeans from Utah (CEU) using reads with evidence of deamination (PMD score 3) and unfiltered reads (PMD score -2), extracted with PMDtools[5].

# Supplementary Figure S7

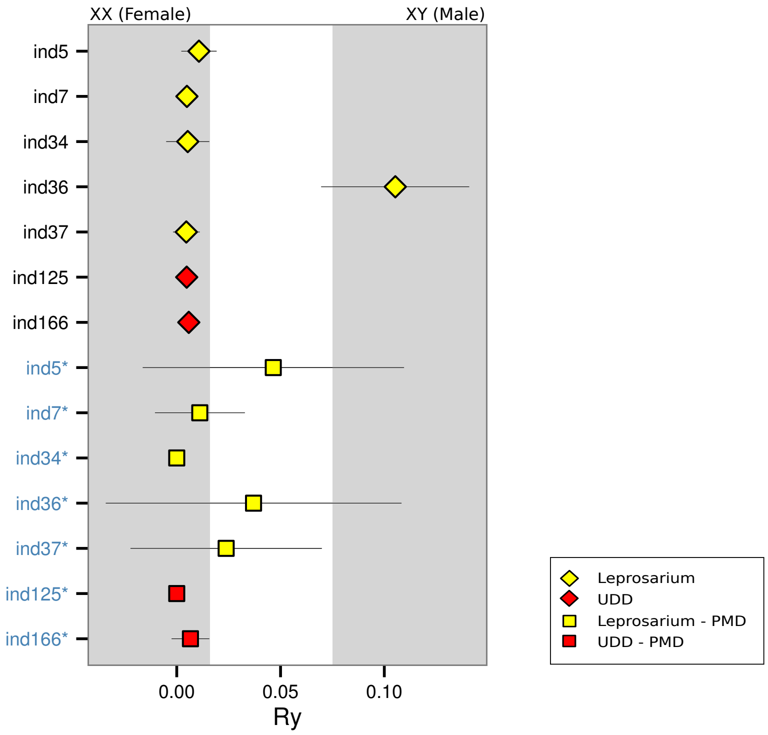


**Supplementary Figure S7 -** Comparison of sex determination[6] results using reads with evidence of deamination (PMD score 3, squares) and unfiltered reads (lozenges). In the analysis using deaminated reads, samples 34 and 125 have 0 reads aligned to the Y chromosome, and therefore confidence intervals for this analysis have not been estimated.

# Supplementary Tables

**Supplementary Table S1** - Sex determination method[6]. CI - Confidence Intervals, SE - Standard Error, R_y – Ratio of the number of reads aligned to the Y-chromsome divided by the sum of the number of reads aligned to the Y- and X-chromosome.

| Sample | Nseqs | NchrY+NchrX | NchrY | R_y | SE | 95% CI | Assignment |
|---|---|---|---|---|---|---|---|
| Ind. 5 | 10740 | 562 | 6 | 0.0107 | 0.0043 | 0.0022-0.0192 | consistent with XX but not XY |
| Ind. 7 | 16300 | 813 | 4 | 0.0049 | 0.0025 | 0.0001-0.0097 | XX |
| Ind. 25 | 799 | 20 | 1 | 0.05 | 0.0487 | -0.0455-0.1455 | Not Assigned |
| Ind. 34 | 3800 | 188 | 1 | 0.0053 | 0.0053 | -0.0051-0.0157 | XX |
| Ind. 36 | 9502 | 285 | 30 | 0.1053 | 0.0182 | 0.0696-0.1409 | consistent with XY but not XX |
| Ind. 37 | 8255 | 431 | 2 | 0.0046 | 0.0033 | -0.0018-0.0111 | XX |
| Ind. 65 | 1657 | 36 | 1 | 0.0278 | 0.0274 | -0.0259-0.0815 | Not Assigned |
| Ind. 125 | 45414 | 2277 | 11 | 0.0048 | 0.0015 | 0.002-0.0077 | XX |
| Ind. 166 | 107507 | 5384 | 31 | 0.0058 | 0.001 | 0.0037-0.0078 | XX |

**Supplementary Table S2** - Sex determination[6] using only reads with evidence of deamination (PMD Score 3), selected using PMDtools[5].

| Sample | Nseqs | NchrY+NchrX | NchrY | R_y | SE | 95% CI | Assignment |
|---|---|---|---|---|---|---|---|
| Ind. 5 | 872 | 43 | 2 | 0.0465 | 0.0321 | -0.0164-0.1095 | Not Assigned |
| Ind. 7 | 2043 | 90 | 1 | 0.0111 | 0.011 | -0.0105-0.0328 | consistent with XX but not XY |
| Ind. 34 | 453 | 25 | 0 | 0 | 0 | 0.0-0.0 | consistent with XX |
| Ind. 36 | 945 | 27 | 1 | 0.037 | 0.0363 | -0.0342-0.1083 | Not Assigned |
| Ind. 37 | 758 | 42 | 1 | 0.0238 | 0.0235 | -0.0223-0.0699 | consistent with XX but not XY |
| Ind. 125 | 2806 | 119 | 0 | 0 | 0 | 0.0-0.0 | consistent with XX |
| Ind. 166 | 6398 | 304 | 2 | 0.0066 | 0.0046 | -0.0025-0.0157 | XX |

**Supplementary Table S3** - Mitochondrial DNA Haplogroups identified with Haplogrep[7]. Accuracy of haplogroup identification is given by Haplogrep Quality. Mutations found in PhyloTree build 16[8] are shown in bold. Italic represents mutations not found in PhyloTree and are likely to be deamination and sequencing errors.

| SampleID | mtDNA reads | Haplogroup | HaploGrep's Quality | Polymorphisms |
|---|---|---|---|---|
| 5 | 11 | H2a2a | 0 | *2094A 2906T* |
| 7 | 12 | L1b1 | 53.2 | **5036G** *6656T* **5046A 357G 9540C** |
| 34 | 3 | - | - | - |
| 36 | 7 | L3i1b | 37.7 | **8860G 13687T 13692T** |
| 37 | 7 | L3'4'6 | 55.9 | **769A 750G 7256T** |
| 125 | 39 | L2b1 | 55.9 | **1018A** *11471T 8294T* **15301A** *11462T* **6026A** *15326G* **5331A 10115C 5814C** |
| 166 | 135 | L3d | 43.4 | **2706G** *1990A* **8860G** *13863T 15714T 14205T 4593T 2637T 1966A 13330T* ***4335T*** *4467T 11658T 13328T 14811T 3430T* ***14766T*** *2670T 14204T 698T 5147A 6541A 9849T* ***15884A*** *13695T 15574T 13916A 14329T 15773A 11714T* ***13886C 9540C 47A*** |

## References


1. Patterson, N., Price, A. L. & Reich, D. Population structure and eigenanalysis. *PLoS Genet.* **2,** 2074–2093 (2006).

2. The 1000 Genomes Project Consortium. An integrated map of genetic variation. *Nature* **135,** 0–9 (2012).

3. Alexander, D. H., Novembre, J. & Lange, K. Fast model-based estimation of ancestry in unrelated individuals. *Genome Res.* **19,** 1655–64 (2009).

4. Li, J. Z. *et al.* Worldwide human relationships inferred from genome-wide patterns of variation. *Science* **319,** 1100–4 (2008).

5. Skoglund, P. *et al.* Separating endogenous ancient DNA from modern day contamination in a Siberian Neandertal. *Proc. Natl. Acad. Sci.* 1318934111– (2014). doi:10.1073/pnas.1318934111

6. Skoglund, P., Storå, J., Götherström, A. & Jakobsson, M. Accurate sex identification of ancient human remains using DNA shotgun sequencing. *J. Archaeol. Sci.* **40,** 4477–4482 (2013).

7. Kloss-Brandstätter, A. *et al.* HaploGrep: a fast and reliable algorithm for automatic classification of mitochondrial DNA haplogroups. *Hum. Mutat.* **32,** 25–32 (2011).

8. Van Oven, M. & Kayser, M. Updated comprehensive phylogenetic tree of global human mitochondrial DNA variation. *Hum. Mutat.* **30,** E386–E394 (2009).
